# Red Chicory (*Cichorium intybus* L. cultivar) as a Potential Source of Antioxidant Anthocyanins for Intestinal Health

**DOI:** 10.1155/2013/704310

**Published:** 2013-08-27

**Authors:** Laura D'evoli, Fabiana Morroni, Ginevra Lombardi-Boccia, Massimo Lucarini, Patrizia Hrelia, Giorgio Cantelli-Forti, Andrea Tarozzi

**Affiliations:** ^1^National Research Institute on Food and Nutrition, Via Ardeatina 546, 00178 Rome, Italy; ^2^Department of Pharmacy and Biotechnology, Alma Mater Studiorum, University of Bologna, Via Irnerio 48, 40126 Bologna, Italy; ^3^Department for Life Quality Studies, Alma Mater Studiorum, University of Bologna, Corso d'Augusto 237, 47921 Rimini, Italy

## Abstract

Fruit- and vegetable-derived foods have become a very significant source of nutraceutical phytochemicals. Among vegetables, red chicory (*Cichorium Intybus* L. cultivar) has gained attention for its content of phenolic compounds, such as the anthocyanins. In this study, we evaluated the nutraceutical effects, in terms of antioxidant, cytoprotective, and antiproliferative activities, of extracts of the whole leaf or only the red part of the leaf of Treviso red chicory (a typical Italian red leafy plant) in various intestinal models, such as Caco-2 cells, differentiated in normal intestinal epithelia and undifferentiated Caco-2 cells. The results show that the whole leaf of red chicory can represent a good source of phytochemicals in terms of total phenolics and anthocyanins as well as the ability of these phytochemicals to exert antioxidant and cytoprotective effects in differentiated Caco-2 cells and antiproliferative effects in undifferentiated Caco-2 cells. Interestingly, compared to red chicory whole leaf extracts, the red part of leaf extracts had a significantly higher content of both total phenolics and anthocyanins. The same extracts effectively corresponded to an increase of antioxidant, cytoprotective, and antiproliferative activities. Taken together, these findings suggest that the red part of the leaf of Treviso red chicory with a high content of antioxidant anthocyanins could be interesting for development of new food supplements to improve intestinal health.

## 1. Introduction

Many degenerative diseases of the gastrointestinal tract, such as colorectal cancer or inflammatory bowel disease, are associated with a persistent state of oxidative stress which results from an imbalance in the production of reactive oxygen species (ROS) and cell antioxidant defenses at intestinal epithelial level [1, 2]. In particular, various exogenous and endogenous sources, including dietary oxidants and inflammatory processes, respectively, together with the disruption of the glutathione antioxidant defense system support oxidative stress within intestinal cells [3, 4]. Recent studies have shown that the induction of oxidative shift in the cellular redox status induces epigenetic events and cellular mitogenic or apoptotic responses which contribute to progression of colorectal cancer [5, 6].

In this context, there is increasing interest in food as a source of antioxidant phytochemicals which can provide an inexpensive readily applicable and easily accessible approach for cancer control and prevention [7, 8]. Several studies support this interest, showing that consumption of fruits and vegetables is associated with a decreased risk of several cancers, particularly colorectal cancer, possibly linked to their phytochemical content, which is of interest due to several proposed health benefits, including antioxidant and anticancer activities [9]. Preserving the redox status of intestinal cells as well as preventing the early events of cellular oxidative damage involved in the carcinogenesis processes by administrating dietary phytochemicals therefore provides an important strategy for colorectal cancer chemoprevention [10].

Among vegetables, chicory (*Cichorium intybus*), a typical vegetable indigenous to Europe and North and Western America, has gained attention for its content of phytochemicals with potential nutraceutical effects, such as phenolic acids, flavonoids, and anthocyanins [11]. Using analytical methods, various studies demonstrate the ability of different chicory varieties to counteract various free radicals, as well as a linear correlation between phytochemical content and antioxidant capacity of this vegetable [12–14]. A more recent study shows an interesting antioxidant activity of the red chicory variety against the oxidative stress response in a eukaryotic model system suggesting its health activity at cellular level [15].

By contrast to other chicory varieties, red chicory is characterized by a high content of anthocyanin pigments [16]. The presence of anthocyanins in red chicory is of special interest because several studies have described many beneficial health or nutraceutical effects of anthocyanins on visual capacity, brain cognitive function, obesity, cardiovascular risk, and cancer prevention [7, 17–19]. The anticancer properties of dietary anthocyanins have been widely evaluated in *in vitro* studies and some animal gastrointestinal cancer models [7]. Although many studies have evaluated the anticancer effects, mainly as antiproliferative activity, of various berry fruit extracts or of anthocyanins from berries in human colon cancer cells, the ability of these dietary phytochemicals to also prevent oxidative events underlying colon cancer in appropriate intestinal models still remains unanswered. An integrated experimental approach with *in vitro* colon cancer and normal intestinal models is needed to characterize the potential nutraceutical effects, including both the prevention and the control of colon cancer, of food or part of a food.

The present study was planned to investigate the levels of phytochemicals, such as total phenolics and anthocyanins, and total antioxidant activity (TAA) as well as the *in vitro* bioactivity of red chicory of Treviso, a typical Italian red leafy vegetables which has been attributed to Protected Geographical Indication status according to European Union rules [20]. The *in vitro* bioactivity, in terms of antioxidant, cytoprotective, and antiproliferative activities, of red chicory extracts was studied using different intestinal models, including human colon carcinoma (Caco-2) cells differentiated in normal intestinal epithelia and undifferentiated Caco-2 cells. In particular, both antioxidant and cytoprotective activities were evaluated in differentiated Caco-2 cells, which provide a suitable model for assessment of the physiological response of intestinal epithelia to oxidative injury [21]. To mimic oxidative damage to intestinal epithelia, we used tert-butyl hydroperoxide (*t-*BuOOH) due to its ability to generate peroxyl and alkoxyl radicals, that catalyze the peroxidation of membrane lipids [22].

## 2. Materials and Methods

### 2.1. Chemicals

Gallic acid, tetrazolium salt (MTT), *t*-BuOOH, 2,2′-azino-bis-(3-ethylbenzothiazoline-6-sulphonic acid) diammonium salt (ABTS), 2′-7′dichlorodihydrofluorescein diacetate (DCFH-DA), and Folin-Ciocalteu's phenol reagent were obtained from Sigma Chemical Co. (St. Louis, MO, USA). All other reagents were of analytical grade purity commercial available.

### 2.2. Sample Selection

Treviso red chicory (*Cichorium intybus* L. cultivar) from certified producers operating in Veneto (Italy) was purchased from a single retail outlet. Four different lots of red chicory at comparable ripening stages were analyzed. All red chicory samples were processed within 48–72 h of purchase.

### 2.3. Sample Extraction

Briefly, 100 g of weight of the whole leaf (WL) or only the red part of the leaf (RL), without the white side, of Treviso red chicory was mixed with cold methanol (HCl 0.1%) and homogenized using an Ultra Turrax homogenizer for 5 min. The mixture was filtered through a Whatman paper under vacuum, and the methanol in the filtrate was evaporated at 35°C. Subsequently, the residue was diluted to 10 mL with methanol and stored at −20°C until phytochemical content analysis. Subsequently, some samples of residue in methanol were dried completely and resuspended in appropriate culture mediums for the bioactivity determinations.

### 2.4. Total Phenolic Content Analysis

Total phenolic concentrations were measured using the Folin Ciocalteau assay [23]. Briefly, appropriate dilutions of extracts were treated with Folin Ciocalteau reagent, and the reaction was neutralized with 7% sodium carbonate. The absorbance of the resulting blue color was measured spectrophotometrically at 750 nm using a Beckman DU 7400 spectrophotometer. Gallic acid was used as standard and results expressed as milligrams of gallic acid equivalents (GAE) per 100 g of the fresh edible part of the red chicory.

### 2.5. Total Anthocyanin Content Analysis

Total anthocyanin content of the extracts of Treviso red radicchio was determined spectrophotometrically by the pH differential method of Rapisarda et al. [24].

### 2.6. Determination of TAA

The TAA of the extracts derived from Treviso red chicory was measured as reported by Re et al. [25]. This method is based on the ability of the antioxidant molecules in the vegetable extracts to reduce the radical cation of the ABTS, determined by the decolorization of ABTS^∙+^ and measured as quenching of absorbance at 740 nm. Values obtained for each sample were compared with the concentration-response curve of a standard Trolox solution and expressed as micromoles of trolox equivalent antioxidant activity (TEAA)/100 g of fresh vegetable.

### 2.7. Cell Cultures of Human Colon Carcinoma Cells

Human colon carcinoma (Caco-2) cells were routinely grown at 37°C in a humidified incubator with 5% CO_2_ in Dulbecco's Modified Eagle's Medium (DMEM) supplemented with 20% (FCS), 2 mmol/L glutamine, 50 U/mL penicillin, and 50 *μ*g/mL streptomycin. To evaluate intracellular antioxidant and cytoprotective activities as well as antioxidant activity in membrane and cytosolic fractions of the WL and RL extracts, Caco-2 cells were seeded at a density of 8 × 10^4^ cells/cm^2^ in multi-well dishes; once the cells were confluent, the medium was changed every 48 h using DMEM 20% FCS. Experiments were performed using completely differentiated cultures at 12–14 days after seeding. To evaluate the antiproliferative activity of the same extracts, undifferentiated Caco-2 cells were seeded in 96-well microliter plates at a density of 1.5 × 10^4^ cells/cm^2^. Experiments were performed after 24 h of incubation at 37°C in 5% CO_2_.

### 2.8. Determination of Intracellular Antioxidant and Cytoprotective Activities

We evaluated the antioxidant and cytoprotective activities of WL and RL extracts of Treviso red chicory against both formation of intracellular ROS and cytotoxicity in differentiated Caco-2 cells after treatment with *t*-BuOOH. Formation of intracellular ROS was determined using a fluorescent probe, DCFH-DA, as described by Wang and Joseph [26]. Briefly, differentiated Caco-2 cells were incubated for 4 h with different concentrations of the extracts derived from Treviso red chicory samples corresponding to 5–30 mg vegetable/mL. Cells were washed with PBS and then incubated with 5 *μ*M DCFH-DA in phosphate buffered saline (PBS) in 5% CO_2_ at 37°C for 30 min. After removal of DCFH-DA and further washing, the cells were incubated with 0.5 mM *t*-BuOOH in PBS for 30 min. At the end of incubation, the fluorescence of the cells from each well was measured (wavelength: 485/535 nm) with a spectrofluorometer (Spectra Max Gemini, Molecular Devices, MN, USA). The values are expressed as percentage of increase of intracellular ROS evoked by exposure to *t*-BuOOH.

Cytotoxicity was monitored by trypan blue uptake as previously described [27]. Briefly, differentiated Caco-2 cells were incubated for 4 h with extracts of WL and RL (corresponding to 5–30 mg vegetable/mL), washed with PBS, and then incubated with 0.5 mM *t*-BuOOH in DMEM 20% FBS at 37°C in 5% CO_2_. After 24 h of incubation, the cells were collected by gentle scraping in PBS and dispersed by repeated gentle pipetting. An aliquot of cell suspension was then diluted 1 : 1 with 0.5% trypan blue in 10 mM sodium phosphate buffer (pH 7.2) and placed on a hemocytometer with a cover slip. Percentages of viable cells were recorded on at least three separate counts.

### 2.9. Determination of Antioxidant Activity in Membrane and Cytosolic Fractions

Cytosolic and membrane-enriched fractions were separated as we previously reported [28]. Briefly, after 4 h of incubation with WL and RL extracts (corresponding to 5–30 mg vegetable/mL) at 37°C in 5% CO_2_, differentiated Caco-2 cells were washed 3 times with cold PBS. Cells were subsequently collected in 1 mL of PBS and centrifuged for 10 min at 10,000 rpm at 4°C, after which the supernatant was removed and the cells were washed with 1 mL of PBS. This was repeated further 2 times, and the pellet was finally reconstituted in 600 *μ*L of 0.05% Triton X-100. Cells were then homogenized and allowed to stand at 4°C for 30 min. Cytosolic and membrane fractions were subsequently separated by centrifugation at 14,000 rpm for 15 min at 4°C. Membrane and cytosolic fractions were stored at −20°C. Small amounts were removed for determination of the protein concentration using the Bradford method. TAA was then measured on cytosolic and membrane fractions using ABTS method as previously reported [25]. This experimental approach makes it possible to determine the cellular uptake of bioactive molecules and their ability to counteract the free radicals at different subcellular levels. Values obtained for each cellular fraction sample were expressed as *μ*mol of Trolox equivalent antioxidant activity per mg of protein.

### 2.10. Determination of Antiproliferative Activity

The antiproliferative activity of WL and RL extracts of Treviso red chicory was determined in undifferentiated Caco-2 cells *in vitro* as we previously reported [21]. Briefly, after 96 h of incubation with WL and RL extracts (corresponding to 1–50 mg vegetable/mL), Caco-2 cells were washed with PBS and then incubated with MTT (5 mg/mL) in PBS for 4 h. After removal of MTT and further washing, the formazan crystals were dissolved with isopropanol. The amount of formazan was measured (405 nm) with a spectrophotometer (TECAN, Spectra model Classic, Salzburg, Austria). The cell viability was expressed as a percentage of control cells. At least three independent dose-response curves were plotted, and the concentration of red chicory extracts resulting in 50% inhibition of cell proliferation (IC_50_) was calculated.

### 2.11. Statistical Analysis

Data are reported as mean ± SD of at least 3 independent experiments. Compositional data were statistically processed utilizing the Student's *t*-test. Statistical analysis of biological data was performed using one-way ANOVA with Dunnett's Post Hoc Test and Student's *t*-test, as appropriate, and Pearson's correlation coefficient for relations among variables. Differences were considered significant at *P* < 0.05. Analyses were performed using PRISM 3 software on a Windows platform.

## 3. Results and Discussion

We first determined the phytochemical contents such as total phenolics and anthocyanins in extracts obtained from edible samples of WL or RL of Treviso red chicory. As reported in Table 1, the extracts of RL had a significantly higher total phenolic and anthocyanin content than the corresponding extracts of WL (all *P* < 0.05). In parallel, the TAA of the same extracts was measured by ABTS radical cation decolorization assay and expressed as *μ*mol of trolox equivalent antioxidant activity (TEAA)/100 g edible sample (Table 1). On the basis of weight, the TAA of the RL extracts was significantly higher than the activity of WL extracts (*P* < 0.05).

Taken together, the high levels of total phenolic and anthocyanin content found in WL extracts are in the range already reported in the literature for other varieties of red chicory [13, 14, 16, 29]. In this regard, we also confirmed the low levels of other antioxidant components such as total ascorbic acid in both WL and RL extracts of red chicory (data not shown). These findings are supported by a recent antioxidant characterization of *Cichorium intybus* that recorded the marginal role of total ascorbic acid in the total antioxidant activity of this vegetable [30]. 

Interestingly, the levels of the same phenolic compounds as well as TAA of RL extracts were significantly higher than WL extracts, supporting the hypothesis that phenolic compounds may be important antioxidant components to account for the observed antioxidant activity. In particular, recent studies on the phytochemical composition of red chicories recorded a large amount of hydroxybenzoic and hydroxycinnamic acids as well as of red anthocyanins which give red chicories an exceptionally high peroxyl radical scavenging activity, in terms of both capacity and efficiency [16]. Although we did not characterize the phenolic compound content, it is reasonable to suppose that the combination of these compounds, mainly the red anthocyanins, could be important due to the higher TAA observed in RL extracts and to confer a potential health value to this part of red chicory.

**Figure 1:**
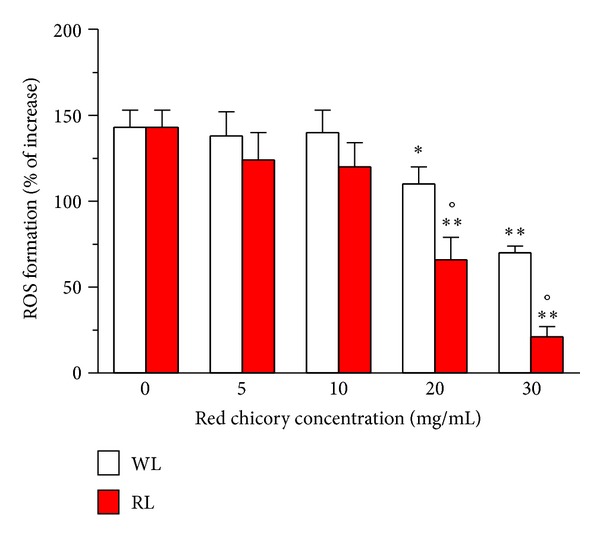
Antioxidant activity of WL and RL extracts of Treviso red chicory in Caco-2 cells differentiated in normal intestinal epithelia. The cells were treated with various concentrations of extracts for 4 h and then treated with *t-*BuOOH (0.5 mM) for 30 min. At the end of incubation, intracellular ROS formation was determined using a fluorescence probe, DCFH-DA, as described in the Materials and Methods section. The values are expressed as percentage of increase of intracellular ROS formation evoked by exposure to *t*-BuOOH. The values are shown as mean ± SD of four independent experiments (*P < 0.05, **P < 0.01 versus untreated cells, at ANOVA with Dunnett's Post Hoc Test; °*P* < 0.05 versus cells treated with WL extracts at Student's *t*-test).

**Figure 2:**
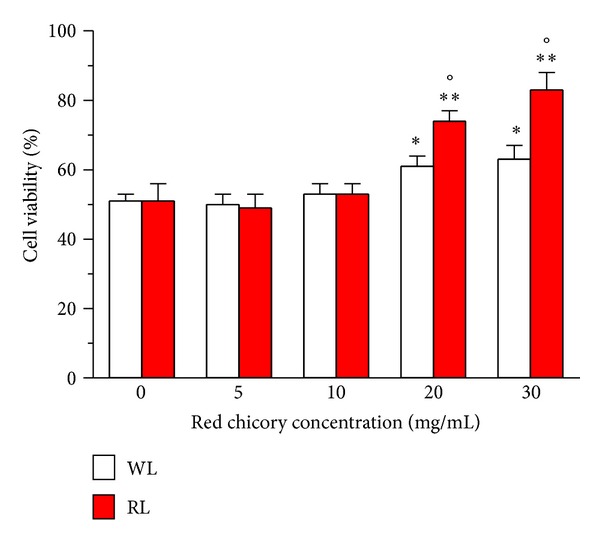
Cytoprotective activity of WL and RL extracts of Treviso red chicory in Caco-2 cells differentiated in normal intestinal epithelia. The cells were treated with various concentrations of extracts for 4 h and then treated with *t-*BuOOH (0.5 mM) for 24 h. At the end of incubation, cytotoxicity was determined using trypan blue assay as described in the Materials and Methods section. The values are expressed as percentage of cell viability after exposure to *t*-BuOOH. The values are shown as mean ± SD of four independent experiments (*P < 0.05, **P < 0.01 versus untreated cells, at ANOVA with Dunnett's Post Hoc Test; °*P* < 0.05 versus cells treated with WL extracts at Student's *t*-test).

We then assessed the nutraceutical effects, in terms of the antioxidant and cytoprotective activities, of these WL and RL extracts of Treviso red chicory against both the intracellular ROS formation and cytotoxicity in differentiated Caco-2 cells after treatment with *t-*BuOOH. As shown in Figures 1 and 2, treatment of differentiated Caco-2 cells with both WL and RL extracts (5–30 mg/mL) showed a decrease, in a dose-dependent manner, of intracellular ROS formation and cytotoxicity elicited by *t-*BuOOH. The ability to counteract the ROS formation and cytotoxicity was significantly higher with RL than WL extracts for the concentrations 20 and 30 mg/mL (all *P* < 0.05).

**Figure 3:**
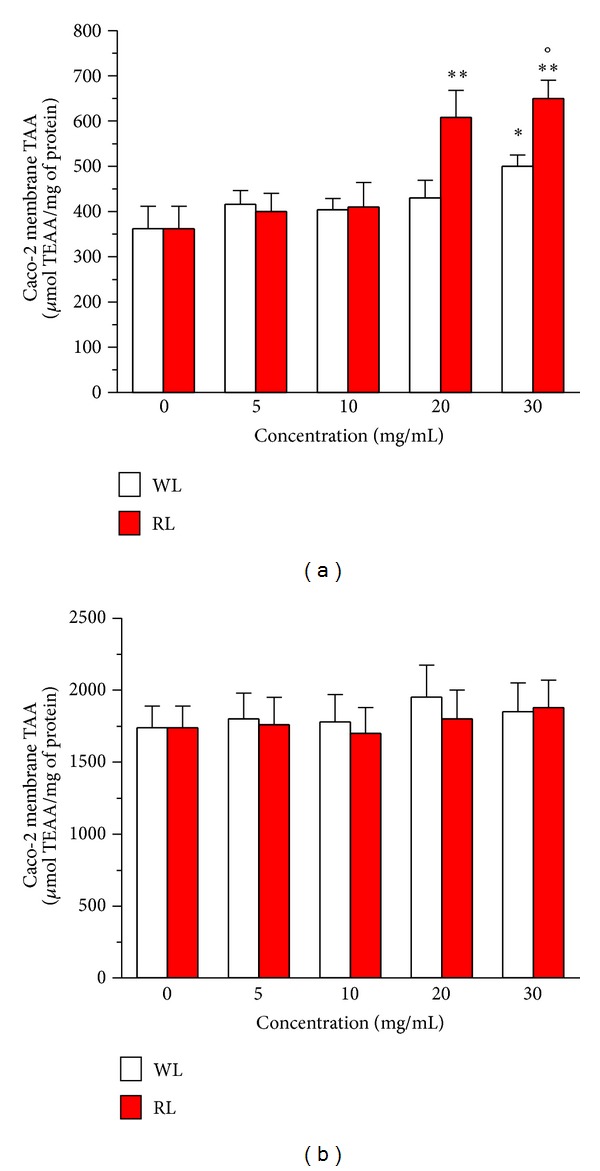
Effects of WL and RL extracts of Treviso red chicory on TAA of membrane (a) and cytosolic (b) fractions of Caco-2 cells differentiated in normal intestinal epithelia. The cellular fractions were submitted to the ABTS methods after 4 h of incubation with various concentrations of extracts as described in Section 2. The results obtained for each cellular fraction sample were expressed as *μ*mol of TEAA per mg of protein. The values represent the mean ± SD of three independent experiments (*P < 0.05, **P < 0.01 versus untreated cells at ANOVA with Dunnett's Post Hoc Test; °*P* < 0.05 versus cells treated with WL extracts at Student's *t*-test).

To confirm the antioxidant and cytoprotective activities of WL and RL extracts at cellular level, we evaluated TAA (expressed as *μ*mol TEAA/mg protein) at two different subcellular levels, membrane and cytosol. As depicted in Figure 3, the membrane and cytosolic fractions obtained from differentiated Caco-2 cells treated with WL (30 mg/mL) and RL (20 and 30 mg/mL) extracts for 4 h showed a significant increase of TAA in comparison with untreated cells. Remarkably, the recorded increase of membrane TAA was significantly higher in Caco-2 cells treated with RL than WL extracts at the concentrations of 30 mg/mL (*P* < 0.05). By contrast, both RL and WL extracts did not modify the basal levels of differentiated Caco-2 cytosolic fraction TAA. Interestingly, a highly significant linear correlation was found between Caco-2 cell membrane TAA and cytoprotective activity for both the WL (*r* = −0.93, *P* < 0.001 at Pearson's correlation coefficient) and RL (*r* = −0.91, *P* < 0.001) extracts.

Taken together, these results show that RL extracts led to greater increases in antioxidant and cytoprotective activities in the differentiated Caco-2 cells than WL extracts. In particular, the highest TAA observed at Caco-2 membrane level could be attributed to higher TAA levels of the anthocyanin fraction of the RL extracts. Our previous studies suggest an accumulation and antioxidant activity of these phenolic compounds present in anthocyanin-rich fruits, such as red orange and strawberry, in the membrane of various human epithelial cells, including intestinal cells [26, 27, 31]. There is evidence that the hydrophobic nature of the ring structure of anthocyanins determines their interactions with the hydrophobic component of the cell membrane, influencing the membrane fluidity and preventing lipid peroxidation and oxidative damage [32]. A recent study supports these scientific considerations, showing the ability of various *Cichorium intybus* extracts to prevent lipid peroxidation and intracellular ROS formation using neuron cell-based assays [33].

**Figure 4:**
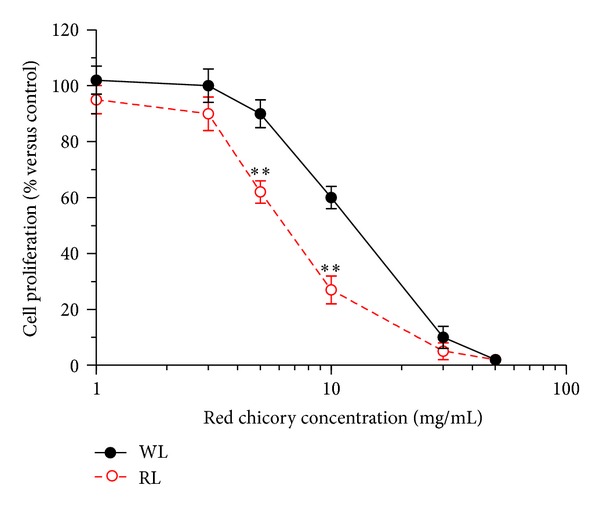
Effects of WL and RL extracts of Treviso red chicory on cell proliferation of undifferentiated Caco-2 cells. The cell proliferation was determined by the MTT assay after 96 h of incubation with various concentrations of extracts. The results were expressed as a percentage of control cells. The values represent the mean ± SD of three independent experiments (**P < 0.01 versus cells treated with WL extracts at Student's *t*-test).

Last, we measured a nutraceutical effect as the antiproliferative activity of the WL and RL extracts in undifferentiated Caco-2 cells using MTT assay. To compare the antiproliferative activity of the extracts, we also used the IC_50_ (concentration of the extract resulting in 50% inhibition of colon cancer cell proliferation) extrapolated by a wide range of extract concentrations, from 1 to 50 mg/mL. As reported in Figure 4, treatment of Caco-2 cells with both WL and RL extracts induced a decrease of cell proliferation in a concentration-dependent manner. The inhibitory effects on Caco-2 cell proliferation were significantly higher with RL than WL extracts for the 5 and 10 mg/mL concentrations (all *P* < 0.01). At higher concentrations, the inhibitory effects were saturated for both extracts. Considering the concentration-inhibitor effect relationship, the IC_50_ was significantly lower with RL than WL extracts (14.85 ± 0.26% versus 21.38 ± 0.47%; *P* < 0.05 at Student's *t*-test).

These findings could be ascribed to a greater amount of bioactive molecules with anticarcinogenicity properties present in red chicory. In this context, it is interesting to note that the *Cichorium intybus* extracts at low concentrations (5 and 10 mg/mL) show antiproliferative effects in the absence of antioxidant and cytoprotective effects. There is an emerging view that phenolic compounds could exert anticarcinogenicity effects not only through their antioxidant potential but also through the modulation of signaling cascades, gene expression involved in the regulation of cell proliferation, differentiation, and apoptosis as well as the suppression of chronic inflammation, metastasis, and angiogenesis [34].

In particular, among phenolic compounds, it has been demonstrated that phenolic acids such as hydroxybenzoic and hydroxycinnamic acids can induce strong antiproliferative effects and apoptosis in human colon cancer cells [9]. Further, several studies also indicate that anthocyanins are able to inhibit the growth of different cancer cells, suggesting their possible role as chemopreventive agents [35, 36]. In particular, it has been suggested that the inhibitory effects of anthocyanin-rich fruit and vegetable extracts are based on the concentration rather than the composition of anthocyanins [37–39]. 

## 4. Conclusions

Our results showed that Treviso red chicory can represent a good source of nutraceutical phytochemicals for intestinal health. In particular, the high levels of antioxidant anthocyanins present in red chicory might exert a direct scavenging effect against ROS formation within the gastrointestinal tract. The anthocyanins and/or their metabolites could further contribute to intestinal health through their ability to spread out in internal intestinal tissue. Recent studies recorded the presence of glycoside, aglycone, and both methylated and glucuronide derivatives of anthocyanins in tissues including the stomach and small intestine of animal models fed either a single anthocyanin or berry extracts [7].

We also demonstrated that the red part of the leaf of red chicory could exert an interesting additional nutraceutical value, in terms of antioxidant and cytoprotective activities as well as antiproliferative activity, with respect to the whole leaf of red chicory. These findings, from an industrial point of view, indicate that the revaluation of some parts of red chicory with a high content of antioxidant anthocyanins could be interesting for the production of new commercial products, such as food supplements of high quality and low cost. However, further clinical studies are needed to confirm whether the whole and the red part of the leaf of Treviso red chicory are likely to improve intestinal health.

## Figures and Tables

**Table 1:** Total phenolics, total anthocyanins, and TAA of edible samples of WL or RL of Treviso red chicory^1^.

Treviso red chicory	Total phenolics^2^	Total anthocyanins	TAA^3^
	mg of GAE/100 g samples	mg of anthocyanins/100 g samples	*µ*mol TEAA/100 g samples
WL	311.6 ± 12.6	110.8 ± 8.2	506.7 ± 35.6
RL	370.4 ± 14.4*	142.6 ± 7.5*	655.2 ± 42.2*

^1^Values are means ± SD of at least four determinations (RL versus WL; *P < 0.05 at Student's *t*-test).

^2^Values were expressed as gallic acid equivalents (GAE) in milligrams per 100 g of edible sample.

^3^Values were expressed as micromoles of trolox equivalent antioxidant activity (TEAA) per 100 g of edible sample.

## Abbreviations

ABTS: 2,2′-Azino-bis-(3-ethylbenzothiazoline-6-sulphonic acid) diammonium salt
Caco-2: Human colon carcinoma cells
DCFH-DA: 2′-7′Dichlorodihydrofluorescein diacetate
GAE: Gallic acid equivalents
MTT: Tetrazolium salt
RL: Red part of leaf
ROS: Reactive oxygen species
TAA: Total antioxidant activity
*t*-BuOOH: *tert*-Butyl hydroperoxide
TEAA: Trolox equivalent antioxidant activity
WL: Whole leaf.
